# Is it time to replace morphine with methadone for the treatment of pain in the neonatal intensive care unit?

**DOI:** 10.1038/s41390-021-01472-z

**Published:** 2021-03-31

**Authors:** John van den Anker

**Affiliations:** 1 Division of Clinical Pharmacology, Children’s National Hospital, Washington, DC USA; 2 Department of Paediatric Pharmacology and Pharmacometrics, University Children’s Hospital Basel, University of Basel, Basel, Switzerland; 3 Intensive Care and Department of Paediatric Surgery, Erasmus Medical Centre–Sophia Children’s Hospital, Rotterdam, The Netherlands

The current prescription opioid epidemic in the USA has also resulted in a growing number of pregnant women and their fetuses being exposed to these opioids. For decades it is known that in utero exposure to both illicit and prescription opioids is associated with an increased risk of preterm birth and can result in neonatal opioid withdrawal syndrome.

Early identification of preterm neonates at risk for the development of severe neonatal opioid withdrawal syndrome in need for pharmacologic treatment, and use of a methadone dosing regimen that can provide effective and safe drug exposure in this vulnerable population have been identified as key factors to assure optimal care of neonates with opioid withdrawal syndrome.

Opioids such as methadone and morphine are used as first-line pharmacologic treatment of neonates with clinical symptoms of opioid withdrawal syndrome.^1^

Methadone is an opioid agonist with a relative long half-life of approximately 25–32 h and is mainly converted by hepatic metabolism via N-demethylation to the inactive metabolite 2-ethylidene-1,5-dimethyl-3,3-diphenylpyrrolidine (EDDP) and subsequently to 2-ethyl-5-methyl-3,3-diphenylpyrroline (EMDP).^2^ It is administered as a racemic mixture of (*R*)- and (*S*)-methadone, although the former enantiomer accounts for most opioid effects and is highly bound to plasma proteins, α_1_-acid glycoprotein being the most relevant one.

Unfortunately, data on the pharmacokinetics and pharmacodynamics of methadone, the most frequently used drug for treatment of neonatal opioid withdrawal syndrome, is still very limited.

A previously published pilot study reported clearance values for term infants after multiple dosing of oral methadone of 8.94 L/h/70 kg.^1^ In addition, Ward et al.^3^ investigated the pharmacokinetics of methadone and its metabolites after intravenous administration and demonstrated formation clearance values of 7.25 and 8.19 L/h/70 kg for *R*- and *S*-methadone to its corresponding metabolites. Large variations in clearance can be observed and might be the result of the variability in the studied neonatal populations (term vs preterm), and route of administration (intravenous vs oral).

Currently, especially information for use of methadone in preterm infants is virtually lacking. This lack of information has resulted in a large variation in dosing regimens used in daily clinical practice.

Moreover, there is a paucity of information concerning the developmental and potential pharmacogenetic aspects of the drug-metabolizing enzymes that are involved in the handling of methadone in these preterm infants that will ultimately determine the exposure to methadone. That lack of information highlights the need for additional studies to investigate the complex pharmacology of methadone and its large inter-individual variability in pharmacokinetics.^4^

Despite this lack of sufficient data on the pharmacokinetics and pharmacodynamics of methadone in neonates, a recent study has shown that short-term outcomes in neonates with opioid withdrawal syndrome were better in those neonates who were treated with methadone as compared to morphine.^5^

As a consequence of that observation one might raise the question if methadone might also be a better choice than morphine for the treatment of neonatal pain in the neonatal intensive care unit, especially in light of the fact that opioids like morphine are not always effective at minimizing pain.^6^ Moreover, the long-term effects of analgesia on neurodevelopment in the presence and absence of pain are still unclear.^7^ Importantly, it is now well documented that painful stimuli in early life result in changes to neurodevelopment lasting into adulthood. Most profoundly, somatosensory processing seems altered, shown as hypersensitivity to re-injury in later life.^8^

It has been shown that neonatal repetitive procedural pain leads to spinal hyperexcitability, expressed as an increased firing of secondary pain transmission neurons.^9^ This increase in firing is possibly linked to *N*-methyl-d-aspartate (NMDA)-receptor-mediated central sensitization lasting into adulthood.^10^ As a consequence, mechanism based analgesia during neonatal pain may prevent these changes. Research shows the early postnatal spinal cord contains functional µ-opioid receptors (MOR) and NMDA-Rs.^11^ Therefore, methadone becomes an interesting candidate for use as analgesic. Methadone has a dual functioning of MOR agonism and NMDA-R antagonism,^12^ which would make it an ideal drug for analgesia and prevention of central sensitization in the early postnatal period.

Van den Hoogen et al.^13^ very recently reported in this journal that neonatal methadone analgesia is able to attenuate acute as well as long-term hypersensitivity associated with neonatal procedural pain in a rat model. In their paper the authors advocate for clinical studies to assess acute and long-term analgesic effectivity of methadone in neonates. They are convinced that these studies can be conducted at this moment because, as is emphasized in both the discussion and conclusion of their paper, not only the pharmacokinetics of methadone in neonates has been published but also the optimal doses of methadone for use in neonates.^3^

Unfortunately, as already mentioned earlier, there is very little information on the pharmacokinetics and pharmacodynamics of methadone in preterm and term neonates. When we look a little bit more in detail at the paper of Ward et al.^3^ we need to realize that only seven neonates (mean postmenstrual age 40 weeks; mean body weight 3.2 kg) were part of this report with a total of 56 infants, children, and adolescents. Furthermore only neonates with a postnatal age of at least 1 week have been included in this paper. It might therefore be too early to initiate clinical studies without having sufficient information on the PK in neonates with different gestational and postnatal ages. The fact that it seemed that neonates had the same clearing capacity as older infants and children might have been simply caused by the relative small number of neonates that were included in this analysis.

Moreover, when we think about treating pain in the neonatal intensive care unit, it is evident that many patients will be preterm infants. It is therefore imperative to have information on how these preterm infants handle methadone before we can initiate clinical trials.

Fortunately, a larger number of preterm infants exposed to oral methadone has been reported by van Donge et al. in 2019.^14^ In that study 31 preterm infants (median gestational age 32 weeks, median body weight 1600 g, and median postnatal age 3 days) were studied and the most important finding was that the clearance of methadone increased with advancing gestational age. The clearance of methadone in these preterm infants increased fivefold between preterm infants with a gestational age of 26 weeks and those with a gestational age of 36 weeks, indicating a major change in these infants as could have been predicted based on the already existing knowledge on maturational changes in the drug-metabolizing enzymes involved in the handling of methadone.

In summary, the research of van den Hoogen et al.,^13^ which was recently published in this journal, provides exciting and stimulating information about the possible use of methadone for the treatment of neonatal pain in the neonatal intensive care unit. It also presents a very compelling case of using mechanism based analgesia during neonatal pain. Based on the data presented it makes complete sense to replace morphine with methadone for the treatment of procedural pain. However, before that move can be done in a scientifically sound way there is an absolute need to determine an optimal dosing regimen for neonates with different gestational and postnatal ages as well as for different routes of administration. To reach that goal there needs to be a collaboration between neonatal medicine specialists, pharmacometricians, and developmental pharmacologists to assure not only the generation of evidence-based data to determine these optimal dosing regimens but also to facilitate the implementation of this new knowledge into daily clinical care in neonatal intensive care units across the globe.
